# Approaching quality improvement at scale: a learning health system approach in Kenya

**DOI:** 10.1136/archdischild-2017-314348

**Published:** 2018-03-07

**Authors:** Grace Irimu, Morris Ogero, George Mbevi, Ambrose Agweyu, Samuel Akech, Thomas Julius, Rachel Nyamai, David Githang’a, Philip Ayieko, Mike English

**Affiliations:** 1 Wellcome Trust Research Programme, Kenya Medical Research Institute (KEMRI), Nairobi, Kenya; 2 Department of Paediatrics and Child Health, University of Nairobi, Nairobi, Kenya; 3 Maternal, Newborn, Child and Adolescent Health Unit, Ministry of Health, Nairobi, Kenya; 4 Kenya Paediatric Association, Nairobi, Kenya; 5 Nuffield Department of Medicine, University of Oxford, Oxford, UK

**Keywords:** quality, low-income country, networks, hospital care

## Background

In 2002, we identified major shortcomings in the management of sick newborns and children at the first referral or district hospital level in Kenya.^1^ Failure in the dissemination of knowledge and skills (and thus of translation of evidence informed policy) was a fundamental problem. To address this challenge between 2005 and 2012 we developed, implemented and studied:

- the national evidence-based clinical practice guidelines in the form of protocol booklets that can be disseminated at scale (and have recently described how this process matured over more than a decade)^2 3^;
- the Emergency Triage Assessment and Treatment plus Admission Care course^4^ (that has been updated over time);
- the standardised medical record forms including checklists of key symptoms and signs that are key elements of the protocols and help define the nature and severity of common illnesses^5^ (also updated over time).

The effect of implementing these tools as part of a multifaceted strategy including outreach, audit and feedback to improve guideline adherence was tested between 2006 and 2009 and proven effective in a cluster randomised trial.^6^ In recent years, we have been able to document wider adoption of the protocols, training and record forms (including uptake outside Kenya) with some evidence of improvements in the quality of district hospital care, measured as adherence to guidelines, beyond centres directly engaged in research.^7–10^

In the last 4 years (2013–2017) we have adopted a new strategy, building on these earlier experiences, to continue efforts to improve hospital care for children in Kenya with a focus on adoption of agreed practice guidelines and uptake of basic technologies. At the heart of this new strategy is a Clinical Information Network (CIN). Here we outline the rationale for and philosophy of the CIN and how we suggest it helps Kenya as a low-income country (LIC) meet Sustainable Development Goals health targets and achieve universal health coverage.^11^ Specifically, we illustrate how the CIN is a mechanism promoting continued improvement of basic hospital services, implementation of new effective practices and technologies, and conduct of locally relevant research to optimise interventions. We argue that each of these issues is often considered a discrete problem, tackled by a ‘confusion’^i^ of programmes or partners. The CIN in contrast is envisaged as a learning health system (LHS) offering a more integrated approach.^12 13^ Key principles of LHS are that they aspire to advance patients’ health through multiple, linked mechanisms including but not limited to:

1. creating a network of engaged and motivated stakeholders involved in the approach’s design, operation and governance;
2. enabling use of information derived from routine clinical data for local improvements and wider health system performance monitoring;
3. promoting more rapid adoption of evidence into routine clinical care;
4. enabling researchers to use the same data to conduct rapid and efficient health research that supports strategic improvements in health.

As a result, LHS are becoming an important part of the healthcare landscape in some high-income countries supporting efforts to maximise efficiency of previously disparate efforts to deliver value in healthcare.^14^

In an earlier report, we have articulated in more detail how adopting the principles of an LHS could provide a framework for productive collaboration between academics, local practitioners and the wider health system aimed at enhancing quality of care in LICs.^12^ Here we reflect on how we have tried to adhere to these tenets of LHS as we have developed the CIN, and explore what we have learnt in the process, while also briefly illustrating some preliminary outputs from work with the Kenyan county hospitals involved in the network. For those interested in a more detailed discussion of the theory of change guiding our approach and the process of its implementation we refer readers elsewhere.^9 15–17^ The work of the CIN has largely focused on improving the adoption of recommended practices by those admitting children to county (formerly district) hospitals in Kenya. These frontline workers are often at pre-registration stage (interns) in Kenya and other LICs. They typically have only 8–10 weeks undergraduate paediatric training. We contend that the ability of this group of frontline workers to ensure that the right treatment is given to the right patients from the onset of their admission is of central importance. In contexts like Kenya, senior clinician review may not occur for many hours and in smaller facilities may never occur.

We organise our reflections on creating the CIN in Kenya around the four key principles of LHS mentioned earlier and offer lessons learnt (summarised in box 1) and examples of challenges (table 1) and achievements linked to each section.

Box 1 **Lessons learnt as part of efforts to adopt the principles of a learning health system**

*Creating a network of engaged and motivated stakeholders in learning system design, operation and governance*

- A history of successful prior engagement with multiple stakeholders that fosters trust between parties is an important foundation supporting local ownership and leadership while it is important that the network addresses each party’s interests.
- Networks are in essence based on continuous communication between parties, the work and importance of communication should not be underestimated.
- Face-to-face meetings remain important to create and sustain individuals’ and institution’s identification with the Clinical Information Network (CIN) and consequently help overcome turnover of specific individuals.

*Enabling the generation and use of local clinical information to promote adoption of better practices and wider health system performance monitoring*

- It is important to develop standardised records and hospital forms through consensus and be sensitive to the realities of routine clinical work processes, existing medical records arrangements and at hospital level how patient files are produced and who pays for them.
- Data can be turned into information that helps support local improvement but such information needs to be credible, timely and appropriate to the users. Investment is required in information use, an area neglected in most low-income countries (LIC), but existing information personnel are keen to support initiatives that recognise the importance of their work.
- There is considerable value in long-term monitoring both locally and in the aggregate as change is often slow and efforts need to be sustained.

*Promoting adoption of basic technologies*

- The ability of senior clinicians and the network to which they belong to influence adoption of basic technologies depends on any initial and recurrent cost and is linked to the number and nature of additional actors required to support adoption.
- Greater future efforts are needed to use information for advocacy to support resource allocation by managers and county and national governments to meet the needs for essential technologies.

*Enabling the conduct of rapid and efficient health research that supports strategic improvements in health*

- Practitioners are interested in contributing to research they see is relevant to their needs and like to be involved in the process through all its stages while acknowledging that specific research skills are needed too.
- A common data resource can enable multiple questions to be addressed efficiently while helping build local research capacity.

**Table 1:** Examples of the challenges encountered during the implementation of the Clinical Information Network related to preservice training, hospital norms and national policies

Level	Examples of challenges
Preservice training	The training of junior medical staff sometimes conflicts with the apparent restrictions on clinical autonomy encompassed by guidelines and their learning experience in tertiary settings where evidence-based guidelines may be looked down on as ‘too simple’. Concerns that increasing student numbers is affecting the quality of undergraduate training exemplified by a paediatrician’s comments: ‘*The interns we are getting are very poor. What I have seen in the last 5 years, I don’t know what kind of training they get.*’ Highly variable graduation dates for clinical cadres within and across institutions challenge planning for orientation of the newly qualified clinicians before their rotation on the wards (and see below). Postgraduate (consultant) training includes little emphasis on management skills such as how to give effective feedback at group or individual levels and in how to foster teamwork among junior clinicians and nurses. Each hospital retained the primary data on its admissions but utilisation of local data by the hospital clinicians and management for more local quality improvement cycles or planning and resource allocation was limited. This was related to relatively poor computer and data analytical skills and limited capacity in hospital records departments with reliance on more intuitive management based on historical contingencies.
Hospital contexts and practical norms	Hospitals often lacked printers/projectors making wide dissemination of feedback reports problematic with a continued reliance on receipt of limited numbers of hard copies. Challenges in local planning and resource mobilisation in hospitals where access to and control over financial resources is very limited. This can impact the continuous supply of hospital stationery such as structured admission (paediatric admission record (PAR)) and discharge forms as well as undermine efforts to improve practices (eg, testing for hypoglycaemia). There is very high turnover of the medical and clinical officer interns such that almost the entire junior clinical team may change every 3 months. Continued role (and in some cases reliance on) of pharmaceutical companies’ support for continued education meetings that may undermine guideline adherence. Practical norms sometimes conflict with practices being promoted such as: that there is little value in writing a short summary of events around the time of death and recording the primary and contributory diagnoses; that ascertaining HIV status should be done by specific HIV counsellors, not clinicians; that anthropometric measurements should be performed by nutritionists, not clinicians; unavailability of some essential services at night and weekends, for example, inaccessibility of special feeds for malnutrition undermining guideline adherence. Epistemic and practical boundaries challenge teamwork. Although audit feedback meetings are intended to be multidisciplinary, they are often cadre-specific undermining efforts at relationship building across cadres and in understanding and tackling system barriers. Competing priorities—the role of heads of paediatric teams is often not perceived as including quality improvement and are given no dedicated time or training for these activities while they also have multiple competing priorities.
National/county governments	Historically weak systems for disseminating policies and monitoring their uptake linked to weak systems for promoting and regulating quality of care. Insufficient capacity for generating and using information as part of decision-making in resource allocation (staff and equipment) to ensure minimum standards of care are achieved.

### Creating a network of engaged and motivated stakeholders in learning system design, operation and governance

We have previously engaged in collaborative research with the Ministry of Health (MoH), the Kenya Paediatric Association (KPA) and the University of Nairobi.^6 18^ The new initiative we describe continued partnerships and extended them to 14 county hospitals (H1–H14).^19^ All these came together to create the CIN focused on inpatient paediatric care. In brief, recruitment of hospitals into the network took place from September 2013 to February 2014. An initial formative event was a consensus meeting to establish the CIN’s vision and mission. It was attended by partner representatives and a focal team from each hospital, comprising a paediatrician, a nurse heading the paediatric ward and the officer in charge of health records and information. This meeting helped align participants’ goals drawing on shared values and resources as part of collective meaning-making and resulted in vision and mission statements.

*CIN vision:* ‘To become leaders in the use of information to improve paediatric hospital care in Kenya and the [East African] region’

*CIN mission:* ‘To generate hospital data we trust to inform our decisions and plans, and monitor and evaluate our actions’

All the partners have remained engaged in the network activities to date. The MoH brings authority, reaffirms policy, helps promote coordination with other programmes, learns about implementation challenges and offers the prospect of improving use of local evidence in policy-making. The KPA provides professional endorsement, helpful in creating a sense of ownership and local identity, as well as being one administrative arm of the partnership. The university and research team currently raise funds (primarily through research grants) and provide expertise in clinical areas, epidemiology/biostatistics, data management and additional project management.^ii^

The CIN focal persons in each hospital are ‘mid-level’ managers who lead departments and teams. In these clinical hybrid roles, they are responsible for improving documentation in medical records that facilitates data collection and ongoing improvement work.^20 21^ The focal persons bring local authority and a distributed form of leadership that promotes CIN legitimacy and credibility. They also understand the local context of hospital systems and power relationships, and play key boundary spanning roles in changing the behaviours of frontline workers that are critical to achieving improvements in care and outcomes.^20 22 23^ Engaging such personnel in improvement work does demand, however, that they have the capability, that the environment offers them the opportunity and that they remain motivated in what can be challenging low-resource settings.^15 24 25^

To help develop ‘engaged and motivated’ members of CIN at local levels and build a collective identity and community of practice, we hold twice yearly face-to-face meetings with paediatricians inviting the other CIN focal persons to one annual meeting.^9^ These meetings allow participants to discuss their audit reports (including offering suggestions for improving data collection) and to identify underlying problems and potential solutions with peers. They also provide a forum for short, specific skills building sessions focused on the ‘soft skills’ needed as a manager (eg, how to run a team) and on building their understanding of research. Crosstalk among the scientific, clinical and policy communities in an arena promoting collegial relationships helps interpret results based on an understanding of practice in the real world. This enables all sides to understand how contexts interact with improvement approaches to bring about observed outcomes^26^ (see box 1 for a summary of lessons learnt).

### Enabling the generation and use of local clinical information to promote adoption of better practices and wider health system performance monitoring

#### Improving information by improving documentation

Many hospitals in LICs have little or no information on the process or outcomes of care, a fundamental challenge to improving quality at scale. A relatively basic system was established as part of CIN so that clinical data are extracted on-site from patients’ paper records at the point of discharge. De-identified data are then collated centrally and a set of routine analytics used to create an audit report for each hospital every 3 months. These reports focus on adherence to guidelines spanning the medical conditions accounting for over 70% of admissions and deaths.^19^ They use simple ‘traffic-light’ coding to identify problem areas and summarise any changes over the previous 12 months in the form of run charts.^16 17^

#### Using data for improvement through regular audit and feedback

**Figure 1:**
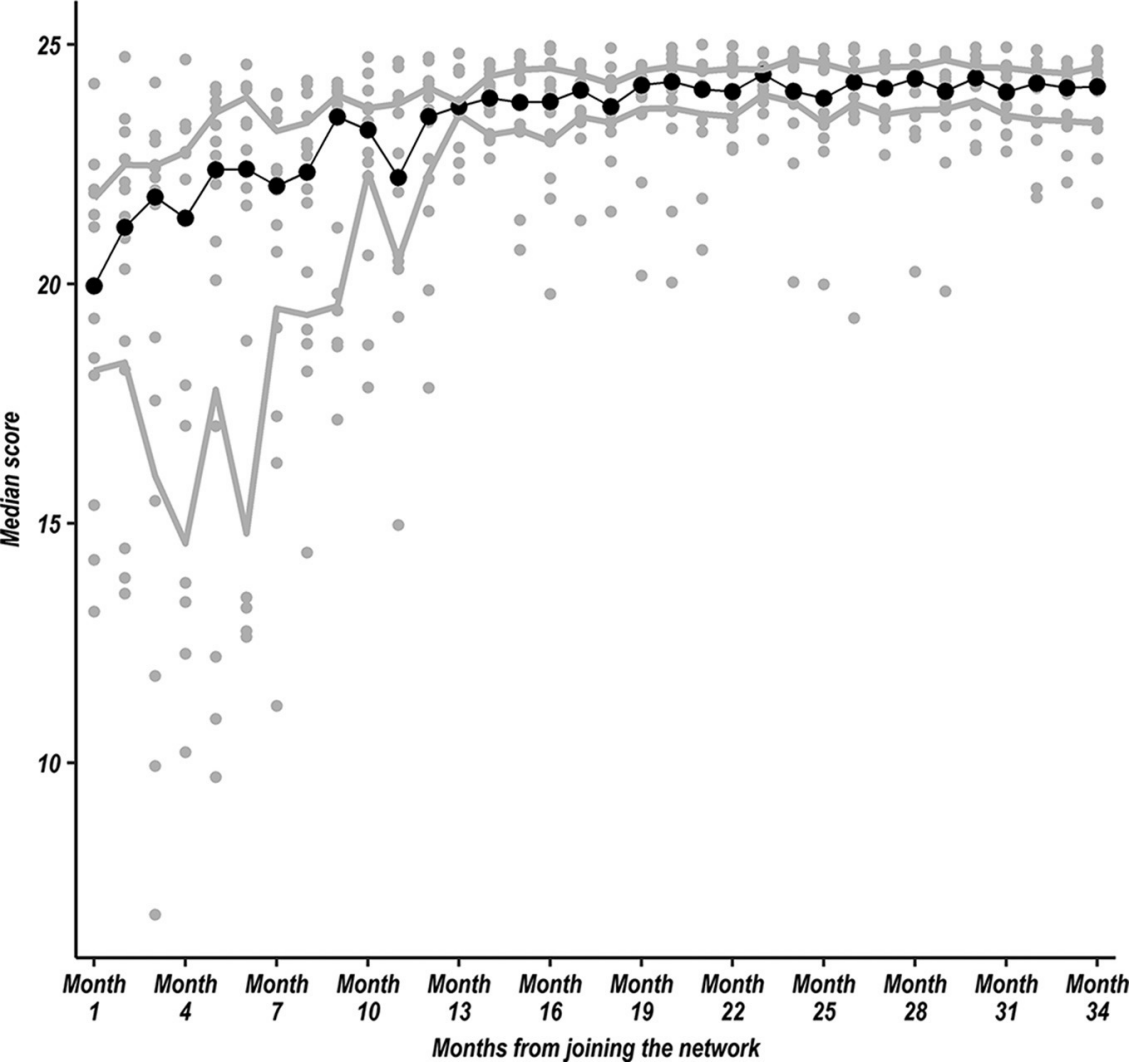
Scatter plot showing each hospital’s performance in documentation (grey circular markers) based on the mean of all patient scores in each month from first month to the 34th month of joining the Clinical Information Network for each site. Each variable (fever, cough, difficulty breathing, diarrhoea, vomiting, convulsions, weight, oedema, stridor, respiratory rate, grunting, chest indrawing, acidotic breathing, wheeze, crackles, temperature gradient, pulse character, capillary refill time, skin pinch duration, sunken eyes, pallor, central cyanosis, disability scale (Alert, Voice, Pain, Unresponsive (AVPU)), ability to drink, stiff neck) is given a score of 1; each patient record is then given a score out of 25 and the mean score calculated for all patients in that month. The solid central trend line with black dots represents the median value of the 14 hospital-specific observations and the upper and lower grey trend lines represent the upper and lower IQRs of the 14 hospital-specific observations, respectively.

Initially we worked on improving documentation in medical records linked to use of an MoH-approved standard paediatric admission record (PAR). Such efforts resulted in an increase in the proportion of children for whom a PAR was used in all hospitals from a median value of 54.05% (IQR 7.55%–92.36%) in the first month of each hospital joining the CIN to 99.47% (IQR 97.99%–100%) by the 34th month. Other approaches included introducing (for the first time) orientation of each new rotation of junior clinicians to preferred practices including use of the PAR on their arrival to the ward. As such rotations occur every 3 months in Kenya, these efforts have likely been critical to sustaining better documentation across more than 3 years (12 rotations) in CIN (see figure 1). To achieve such improvements, we first worked with paediatricians to ensure they could interpret the audit reports and give effective feedback to their hospital teams. Between network inception and November 2016 hospitals have received 15 feedback reports. We observed that hospital teams are keen ‘not to be in the red’ but ‘be green’ in their report. Comparison of hospitals’ performance especially during face-to-face meetings helped foster shared learning rather than potentially harmful competition and helped people gain confidence in their ability to achieve change. We attribute this to having a shared vision and referring to poor performance as ‘offering opportunity for learning and improvement’ while engagement with peers provided encouragement to complete quality improvement processes.^25^ As a result, clinical teams worked with their records department and hospital administration, drawing lessons from across CIN, to ensure hospitals acquired infrastructure, reorganised service delivery and leadership provided effective mentorship and supervision to support delivery of quality care.

**Figure 2:**
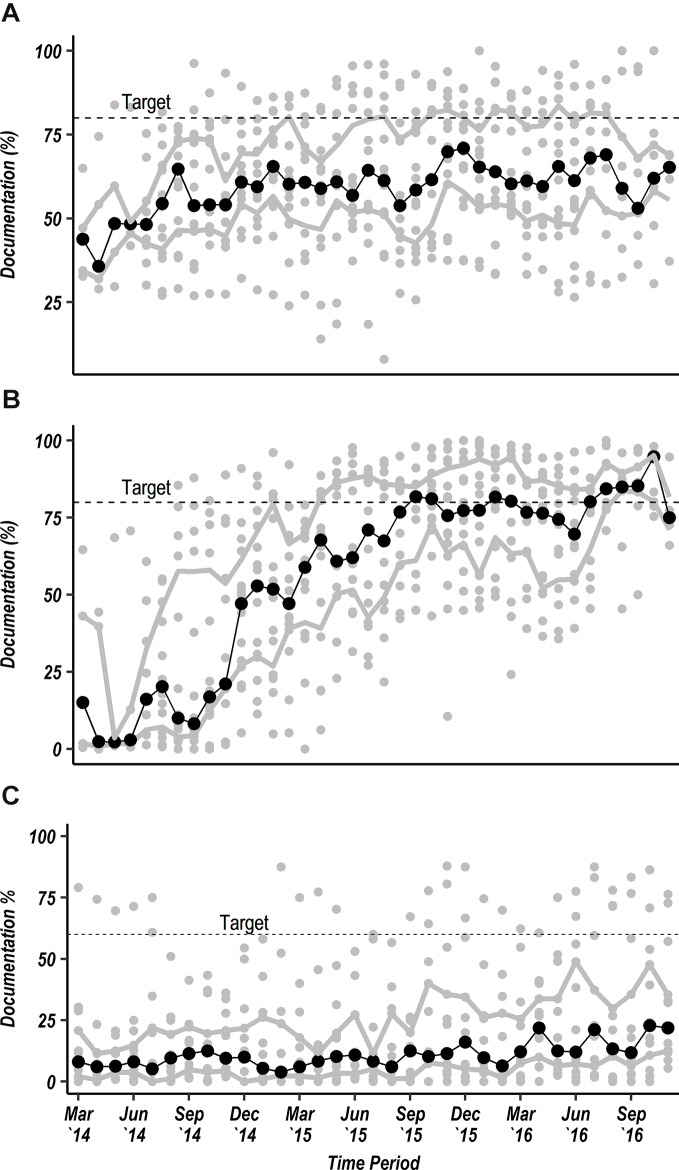
Scatter plots showing each hospital’s performance in documentation (grey circular markers) each month from March 2014 to November 2016 for a clear primary discharge diagnosis for ages 0–12 years (A) and HIV status for all admissions aged 0–12 years (B) both with target documentation rate at 80%. Panel (C) illustrates documentation of blood glucose test results for all patients aged 0–12 years with any danger sign with target of 60%. The solid central trend line with black dots represents the median value of the 14 hospital-specific observations and the upper and lower grey trend lines represent the upper and lower IQRs of the 14 hospital-specific observations, respectively.

From June 2014, feedback included tracking of multiple indicators of adherence to key guidelines and adoption of basic technologies. At a CIN meeting held in October 2015, hospitals together set specific shared targets for three indicators in which it was felt inadequate progress was being made: (1) to improve the documentation of a clear primary discharge diagnosis to 80%; (2) to clearly determine and then document HIV status in records for 80% admissions; and (3) to document blood glucose test results for at least 60% of children admitted with danger signs. There was some improvement in documenting a clear discharge diagnosis (figure 2A) but performance was less good (data not shown) in the subpopulation who died as this required a clear summary of the death to be recorded in the medical file, something not previously practised. Ascertaining HIV status improved over time with seven hospitals achieving the >80% target and a status ascertained for 81.9% of all children in CIN by November 2016 (figure 2B). There was, however, only a small improvement in recording blood glucose levels overall in seriously ill children although two hospitals achieved the >60% target (figure 2C). Informal discussions with CIN focal teams suggested this was in many cases linked to difficulties in securing adequate supplies for bedside or laboratory-based glucose testing. Lack of resources then undermined clinicians’ motivation to request the test.

### Promoting adoption of basic technologies

**Figure 3:**
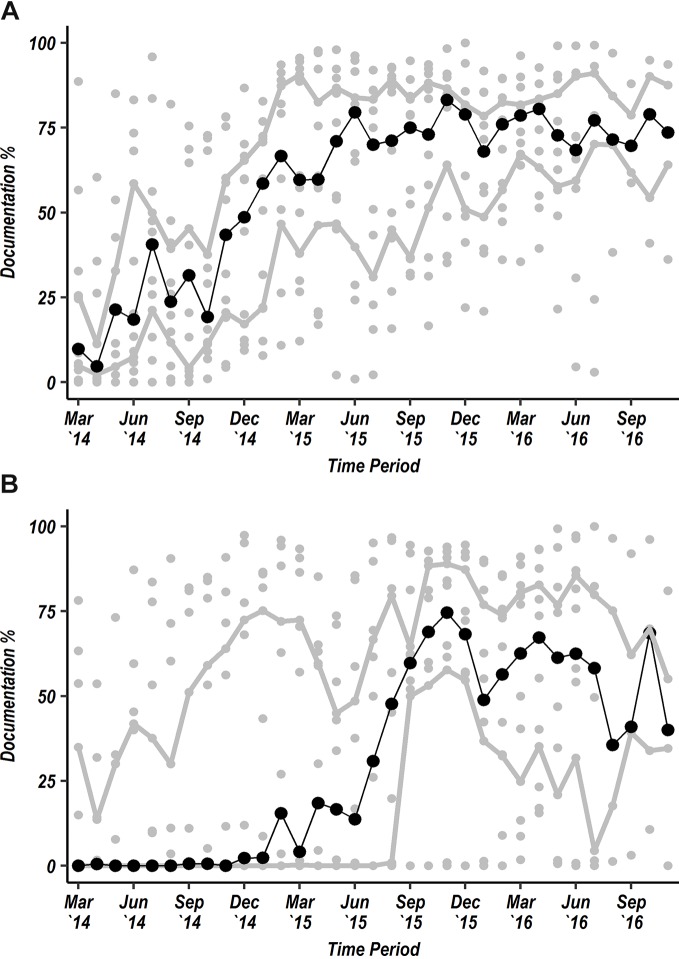
Scatter plots showing each hospital’s performance in documentation (grey circular markers) each month from March 2014 to November 2016 for documentation of mid-upper arm circumference (MUAC) for all admissions aged 6–59 months (A) and documentation of oxygen saturation of all admissions aged 1 month to 12 years (B). The solid central trend line with black dots represents the median value of the 14 hospital-specific observations and the upper and lower grey trend lines represent the upper and lower IQRs of the 14 hospital-specific observations, respectively.

In 2013, the Kenya national guidelines adopted mid-upper arm circumference (MUAC) as the preferred measurement to assess acute malnutrition for children aged 6–59 months. Initial data from CIN demonstrated limited MUAC measurement and MUAC tapes were often lacking. CIN partners therefore lobbied the national Unicef offices, then supplying MUAC tapes for use in community and primary care, to supply tapes to CIN hospitals in April 2014. By November 2016, 71.5% of admitted children had a MUAC measure recorded (figure 3A). Having quality hospital data also enabled some hospitals to negotiate for better supply and use of pulse oximetry. Three hospitals had pulse oximeters at the start of the project and 12/14 by the end of 2016 at which point 49% of all admitted children had oxygen saturation recorded (figure 3B). However, as mentioned above there was less success in increasing measurement of blood glucose (figure 2C) suggesting that efforts must be made by CIN to advocate at local and national levels for improved resources for essential interventions, diagnostics and technologies.

### Enabling the conduct of rapid and efficient health research that supports strategic improvements in health

Most published research from LICs is conducted in specifically resourced research units. The CIN partnership aimed to engage those working in routine settings in research hoping this would help set a relevant research agenda and improve generalisability of research. At the same time, we wished to improve the understanding of research and its value in addressing practical clinical and implementation questions. This viewpoint recognises that research and its translation is not one person or group’s activity but should be viewed holistically as embedded in a system where multiple contributors have inter-related roles.^27^

Between September 2013 and November 2016, basic data were collected from 95 380 paediatric admissions and in 72 355 (75.6%) comprehensive data spanning multiple clinical, treatment and outcome variables were collected. We have used the CIN database for a variety of locally led research reports. These include: (1) developing a better understanding of the challenges facing hospitals and variation in mortality and morbidity^7 19^; (2) addressing local concerns that too many children might be getting harmful fluid boluses for shock management (we found no liberal use of boluses)^28^; (3) evaluating adoption of specific treatment recommendations for severe malaria and respiratory infections^8 29^; and (4) identifying how delays in providing blood transfusion, a system level challenge, increase mortality.^30^

The CIN framework has also provided the platform for a pragmatic, cluster randomised trial of 12 CIN hospitals to test the effects of different feedback strategies on adoption of new pneumonia case management guidelines,^31^ comparative effectiveness analyses of alternative antibiotic regimens for the treatment of pneumonia^32^ and risk factors for mortality from pneumonia.^33^ In direct response to clinician’s concerns the CIN has also conducted an audit across hospitals on the diagnosis, treatment and outcomes of neonatal dehydration,^34^ a topic for which there is no clear international or national clinical guidance. An important additional benefit of the CIN has been its ability to support capacity development in research benefitting five Kenyan PhD students.

## Summary and lessons learnt

Throughout this manuscript we have reflected on simple lessons learnt. We summarise further insights on challenges in table 1 (linked to preservice training, hospital norms and the national context) and lessons learnt in box 1.

Sustaining the type of network we describe requires resources. At hospital level, the network supports a data clerk with no other financial or material support.^9^ Centrally, a dedicated data management and clinical team needs to be supported, as do face-to-face meetings (twice yearly) that are important to building the partnership and sharing learning. While such networks receive support in high-income settings, partnerships focused on the difficult day-to-day work of changing routine practices over extended periods are not often a priority for those supporting programmes or research in LICs. In these settings, partners seem to prefer development of ‘quick technological fixes’ (eg, UKAid’s £16 million Kenyan County Innovation Challenge Fund^35^) with little regard to whether systems can absorb and implement innovations successfully(Indeed, hospitals’ inability to support blood glucose testing in very sick children suggests absorption of technologies at scale remains a major challenge). Many approaches still therefore seem to treat health systems as simple production systems that can be transformed by ‘magic bullets’ rather than recognising that causes of suboptimal performance often requires long-term institutional and individual behaviour change.^25^ Achieving long-term change may require long-term partnerships, something hard to reconcile with often short-term international funding and political horizons.

Additionally, those that initiate (or fund) programmes aimed at transforming care or improving quality often expect implementation of a package of predefined interventions in keeping with a logical framework where cause and effect are linearly linked. However, it is increasingly realised that we are intervening in complex adaptive systems.^36^ Here success may be linked to building of ‘soft skills’ among the CIN focal persons in leading multidisciplinary teams, greater reflection on the dynamic process of intervention with flexibility to learn and amend intervention strategies built into change efforts.^36 37^ We feel that network approaches incorporating the principles of LHS are suited to the complex process of delivering the large-scale, long-term improvements required in hospital care for children in LICs including Kenya.^26^

## Footnotes

i Here we adopt the word confusion, the collective noun for a crowd of the brightly coloured birds guinea fowl, as a metaphor.

ii It is worth noting that other than supporting a data clerk at each hospital to collect data, hospitals receive no other financial or material resources from the network.
